# Development and psychometric validation of an educational support assessment scale for novice home healthcare nurses

**DOI:** 10.1186/s12909-023-04313-1

**Published:** 2023-05-10

**Authors:** Yoko Mori, Miki Sasaki, Yasuko Ogata, Taisuke Togari

**Affiliations:** 1 Department of Gerontological Nursing and Healthcare Systems Management, Graduate School of Health Care Sciences, Tokyo Medical and Dental University, 1-5-45, Yushima, Bunkyo-ku, Tokyo, 113-8510 Japan; 2 Human Life and Health Sciences, Graduate School of Arts and Sciences, The Open University of Japan, Chiba-shi, Japan

**Keywords:** Home healthcare nursing, Novice home healthcare nurses, Scale development, Reliability, Validity

## Abstract

**Background:**

Educational support quality is an essential factor in determining the education of novice home healthcare nurses. This study developed a scale to assess the educational support provided by home healthcare agencies among novice home healthcare nurses.

**Methods:**

Hypothetical components were derived from a literature review, including experiential learning theory. Expert panels evaluated the initial scale items, and the scale was tested with 3000 agencies from April to June 2022. A total of 627 valid responses were analyzed.

**Results:**

Exploratory factor analysis produced a four-subscale structure consisting of 34 items that supported the hypothesized components. Cronbach’s alphas ranged 0.889 to 0.961, and the intraclass correlation coefficients ranged 0.703 to 0.905 in the test-retest survey.

**Conclusions:**

The educational support assessment scale developed for novice home healthcare nurses is valid and reliable. Managers in home healthcare agencies should apply the results of assessments using the scale to improve their human resource development.

**Supplementary Information:**

The online version contains supplementary material available at 10.1186/s12909-023-04313-1.

## Background

Home healthcare is an accepted strategy worldwide for responding to increased healthcare needs, such as a doubled life expectancy in most developed countries [1] and the global burden of late-life diseases [2]. Home healthcare can also be an integral component of the post-hospitalization recovery process, especially during the initial weeks after discharge when the patient still requires some level of regular physical assistance. Home healthcare is usually less expensive, more convenient, and as effective as the care received in a healthcare facility [3]. However, there is a global home healthcare nursing shortage, and the growing need for home healthcare nursing is not being addressed [4, 5].

In Japan, the number of home healthcare agencies is increasing to meet the growing demand for home healthcare nursing; however, the number of home healthcare nurses is still insufficient [6]. Educational support is provided by home healthcare agencies without considering the individual abilities of novice home healthcare nurses [7]. Improving the support provided by home healthcare agencies at the time of employment may lead to early adaptation to the new home healthcare setting. Therefore, it is essential to enhance educational support for novice home healthcare nurses and to retain home healthcare settings. Retaining home healthcare nurses in home healthcare settings and reducing turnover are essential for maintaining a sufficient number of nurses.

Approximately half of the home healthcare nurses in Japan are in their 40s, and the average length of clinical nursing experience is 22 years. Most nurses are engaged in home healthcare settings as a second career after working at medical institutions, such as hospitals [8]. Thus, they need to develop the necessary skills for home healthcare nursing based on the values and experiences they have developed as clinical nurses. However, owing to a shortage of human resources, many small establishments currently have insufficient time to provide educational support for novice home healthcare nurses, including on-the-job training. The primary method of training is the oral transmission of skills, indicating there is no specific educational curriculum [9].

Educational support is defined as providing on-the-job training in accordance with the individual educational needs of novice home healthcare nurses and helping them develop mastery in supporting patients, while also providing feedback concerning their home healthcare nursing experiences. Previous studies reported that nurses recently employed in this field find practice difficult, and that they desire educational support; however, many choose to leave their positions owing to these hardships and unmet support needs [10].

Currently, to our knowledge, no scale has been specifically designed to assess the educational support provided by home healthcare agencies to home healthcare nurses. Though the Practice Environment Scale of the Nursing Work Index, used in many countries, can measure the support and leadership that organizations provide to nurses [11], this scale was primarily developed for hospital settings. Ogata et al. [12] also developed a scale to measure the practice environment in a home healthcare setting, but it was not focused on educational support. The Dundee Ready Education Environment Measure (DREEM) is a tool to assess the educational environment of medical schools and other health training settings. This scale was not developed specifically for home healthcare nurses. In addition, the DREEM subscales, such as Students’ Perception of Learning, measure medical students’ cognition. Therefore, the DREEM was not considered appropriate for our study context.

Our study purpose was to develop an assessment scale—the Educational Support Assessment Scale for Novice Home Healthcare Nurses (ESA-NHHN)—to not only support the retention of nurses in home healthcare settings but also promote their success.

## Methods

### Hypothetical components

**Fig. 1:**
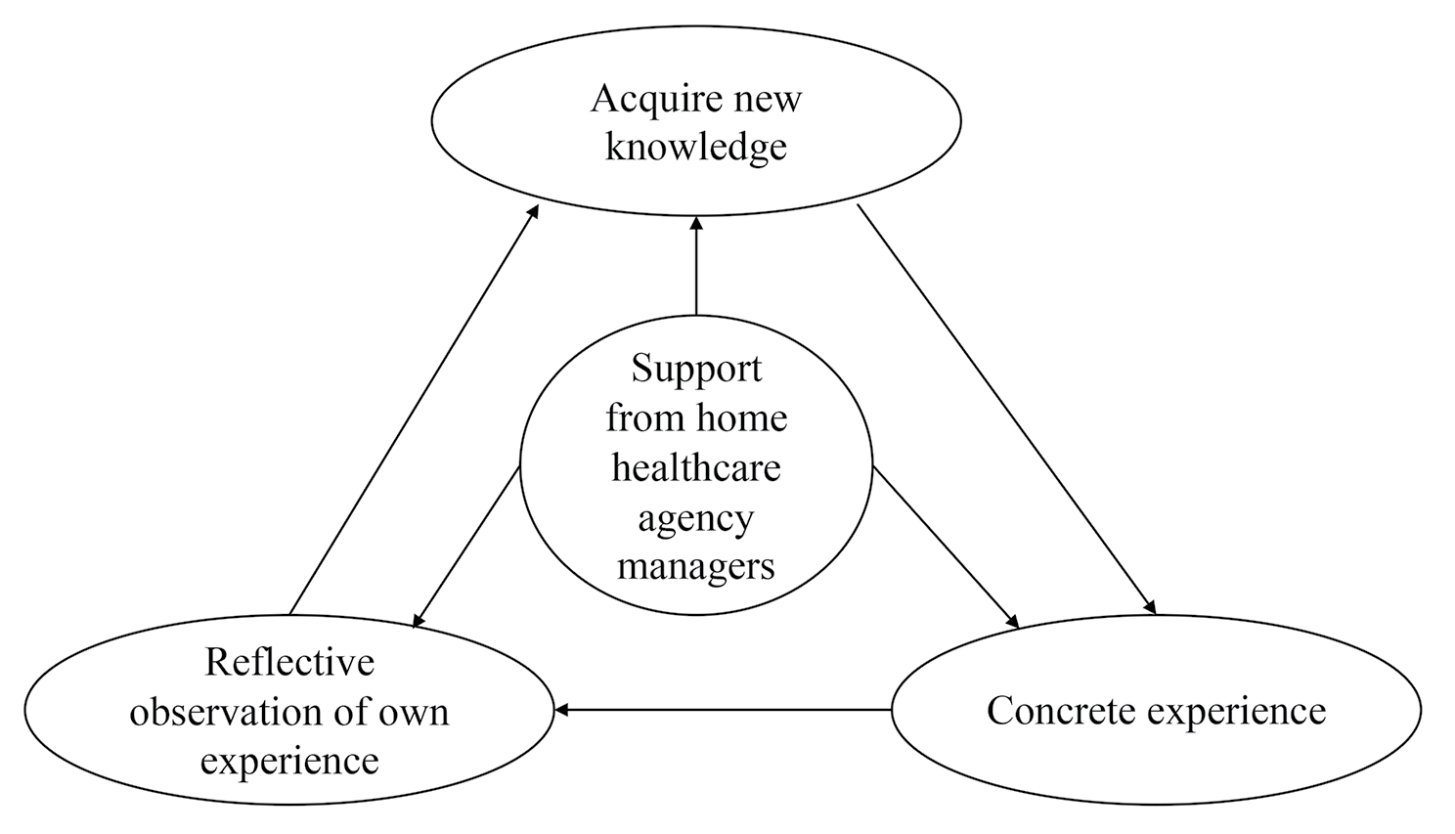
The hypothetical components of Educational Support Assessment Scale for Novice Home Healthcare Nurses (ESA-NHHN)

The hypothetical components of the ESA-NHHN were developed from a comprehensive literature review, including a reference to Kolb’s experimental learning theory. This theory defines learning as “the process whereby knowledge is created through the transformation of experience. Knowledge results from the combination of grasping and transforming experience” [13]. The theory portrays two dialectically related modes of grasping experiences—concrete experience and abstract conceptualization—and two dialectically related modes of transforming experience—reflective observation and active experimentation [14]. Novice home healthcare nurses have adapted to the home healthcare setting by developing their own view of nursing through on-the-job and other training, based on the view of nursing developed through their work experience in medical institutions such as hospitals. To improve learning, the focus should be on engaging students in a process that facilitates optimal learning. This includes providing feedback on the effectiveness of students’ learning efforts [15]. The hypothetical component comprises four elements: “acquire new knowledge,” “concrete experience,” “reflective observation of own experience,” and “support from home healthcare agency managers” (Fig. 1).

### Development of the initial items

Forty-one initial items were created based on an extensive literature review. The literature used to develop the initial items was extracted by two reviewers separately using the same search formula and integrated to ensure reproducibility (Additional file 1). In the review, semi-structured individual interviews were included, conducted by the first author, to identify educational support provided by home healthcare agencies that were positively received by home healthcare nurses when starting a new home healthcare carrier [16]. Fourteen subcategories were extrapolated from the interviews.

### Evaluation of content validity and revision of the initial items

Five clinical experts with experience in the home healthcare setting and who had either a master’s or Ph.D. degree in nursing were asked to evaluate the initial items as an expert panel. They were asked to identify initial items that were unclear, should be revised, or were incompatible with the research objectives. They were also requested to provide any general comments or concerns and evaluate each item using a four-point scale (1 = item is not relevant, 2 = item is somewhat relevant, 3 = item is almost relevant, and 4 = item is relevant). The content validity index (CVI) was calculated to quantify the degree of evaluation using the CVI approach outlined by Lynn [17] and expanded by Polit et al. [18]. Scores lower than 0.78 should be reconsidered through revision, deletion, or substitution [18]. Two items were deleted because their CVI was less than 0.78. Further, we conducted a 90-minute interview with a manager with extensive experience at a home healthcare agency to obtain her opinion on the initial items to enhance their content validity.

While our five clinical experts had experience working in home healthcare settings, we also wanted to include someone who was currently in an educational or research position. Therefore, we sought the advice of a nurse employed in the clinical field with managerial experience at a home healthcare agency who was also a representative of a home healthcare committee. Consequently, two items were added. This led to the identification of 41 ESA-NHHN items.

### Study design

This study utilized a cross-sectional design.

### Participants and procedure

A total of 627 home healthcare nurses participated in the study. Novice home healthcare nurses were defined as nurses who were working for the first time in a home healthcare setting and had previous experience working in a hospital or other medical institution. The inclusion criteria were home healthcare nurses employed for more than six months and less than three years by home healthcare agencies. Nurses who had worked at other home healthcare agencies or newly graduated nurses who had never worked at hospitals or other medical institutions were excluded. For the responses to the initial survey items, we noted that those with less than one year of experience should recall from the time they started until now, while those with more than one year of experience should recall their first year of experience. Therefore, the variation in results is one year. To circumvent recall bias, the maximum number of years of home healthcare nursing experience was set at less than three years.

Participants responded to the questionnaire by recalling the educational support they received from the home healthcare agency where they were currently employed during the first year of employment. Home healthcare nurses were recruited using a publicly available database belonging to the National Association for Visiting Nurse Service as regular members. Equal weightage was given to all home healthcare agencies in the country, and 2700 agencies were randomly selected. Three questionnaires were mailed to each of them. To confirm the test-retest reliability, an additional 300 home healthcare agencies were sent an enclosed cover letter requesting responses to the same questionnaire two or three weeks later. Exploratory factor analysis (EFA) requires a sample size of five to ten times the number of items [19]. Since there were 41 initial items, the number of samples required for EFA was set at 410, and the response rate was estimated at 5%. Thus, the number of questionnaires to be distributed was set at 9000. Since the response rate tends to be low in surveys of home healthcare agencies in Japan, this number was deemed appropriate to ensure a sufficient sample size for factor analysis [20]. Data were collected from April to June 2022.

### Measurements

The questionnaire included the ESA-NHHN, and psychological variables were measured to investigate concurrent validity. Demographic variables, including age, gender, work status, period of working as a clinical nurse, period of working as a home healthcare nurse, the family constitution of the person who required caregiving, and having underage children were collected.

#### The ESA-NHHN

The ESA-NHHN included 41 items, such as “There are opportunities to reflect on one’s own nursing home visits through discussion with colleagues.” Each item was rated on a five-point Likert scale (1 = *strongly disagree*, 5 = *strongly agree*), with higher scores indicating greater educational support by home healthcare agencies among novice home healthcare nurses.

#### External criteria for examining concurrent validity

The ESA-NHHN was developed with the expectation that it would promote nurse retention. Therefore, by examining the relationship between the ESA-NHHN and psychological variables, such as affective commitment, work engagement, job satisfaction of home healthcare nurses, and satisfaction with their home healthcare agencies as external criteria, we confirmed concurrent validity. Since the ESA-NHHN comprises items about the educational support that novice home healthcare nurses should obtain when entering employment, it is expected that there is a theoretical relationship between the ESA-NHHN and external criteria. Receiving appropriate educational support offerings enhances nurses’ affective commitment, such as their sense of belonging to the organization [21]; further, feeling enough job satisfaction encourages retention of employment in the organization [22]. Work engagement is defined as a positive, fulfilling, work-related state of mind characterized by vigor, dedication, and absorption [23]. From these theories, a significant positive correlation is expected. Based on these previous findings, we adopted them as external criteria and examined their relevance.

#### Affective commitment

Affective commitment was assessed using the Affective Organizational Commitment Scale [24], which comprises three items. Items were scored using a five-point Likert scale (1 = *no*, 2 = *most likely no*, 3 = *neither*, 4 = *most likely yes*, 5 = *yes)*, with higher scores indicating higher nurses’ affective commitment to their organization.

#### Work engagement

Work engagement was assessed using the Japanese version of the Utrecht Work Engagement Scale, which is reliable and valid [25]. The items were rated on a seven-point Likert scale (0 = *never*, 6 = *always*), with higher scores indicating higher work engagement.

#### Job satisfaction of home healthcare nurses

Job satisfaction of home healthcare nurses was measured using the Japanese version of the Home Healthcare Nurses’ Job Satisfaction Scale (HHNJS-J), developed by Mori et al. [16], based on the original scale developed by Ellenbecker et al. [26]. The questionnaire included 26 items that represented the seven subscales. Items such as “Patients are satisfied with the care that I provide” were rated on a five-point Likert scale (1 = *strongly disagree*, 5 = *strongly agree*), with higher scores indicating greater job satisfaction. The reliability and validity of the HHNJS-J have been confirmed [27].

#### Satisfaction with their home healthcare agencies

Two original items were created for this study: “I feel I received educational support in adjusting to home healthcare at this home healthcare agency” and “I feel that the manager of this healthcare agency is a good manager and leader.” They were scored using a seven-point Likert scale (1 = *strongly disagree*, 7 = *strongly agree*), with higher scores indicating greater satisfaction.

### Data analysis

Descriptive statistics were calculated for participants’ sociodemographic characteristics.

For the ESA-NHHN, the item analysis was confirmed by checking the ceiling effect (mean + SD > 5), floor effect (mean – SD < 1), collected item-total correlation coefficients (r < 0.3), and the good-poor (G-P) analysis of each item was calculated [28, 29]. Cronbach’s alpha was calculated for the ESA-NHHN for the overall scale score and the four subscales to assess internal consistency. Alpha coefficients greater than or equal to 0.70 were considered satisfactory. To examine test-retest reliability, we used the intraclass correlation coefficient (ICC) of the ESA-NHHN on the four subscales and the overall scale score. An ICC greater than 0.70 was considered satisfactory [30].

EFA was performed to verify the structural validity. The Kaiser-Meyer-Olkin index (KMO) of sampling adequacy and Bartlett’s chi-square test of sphericity (p < 0.05) were employed to confirm the suitability of the data for EFA. KMO was compared to the adequacy of standards (0.80 < meritorious) [31]. The maximum likelihood method with Promax rotation was used for factor extraction because of the assumption that factors were correlated with each other. The number of factors was determined based on scree plots.

Confirmatory factor analysis (CFA) was performed to test the subscale structure extracted from the EFA. Model fit was assessed using a combination of indices, including the goodness-of-fit index (GFI), comparative fit index (CFI), Tucker-Lewis index (TLI), and root mean square error of approximation (RMSEA). For the first two indices, values exceeding 0.90 were considered adequate [32, 33], and for the RMSEA index, values between 0.05 and 0.08 indicated a reasonable fit [34, 35].

Partial correlation coefficients were examined with age as a control variable between the total score and four subscales of the ESA-NHHN and the job satisfaction of home healthcare nurses and other psychological variables to evaluate predictive validity. Partial correlations were classified as poor (< 0.40), moderate (0.40 to 0.70), or strong (> 0.70) [36].

All statistical analyses, except for CFA, were performed using SPSS 26 for Windows (IBM Corp., Armonk, NY, USA). CFA was conducted using AMOS version 26 for Windows (IBM SPSS Statistics Program, Chicago, IL, USA). Statistical significance was set at p < 0.05.

## Results

### Participants’ characteristics

A total of 731 participants returned the questionnaire (response rate = 8.1%). Participants who failed to provide consent or whose responses to the questionnaire were incomplete were excluded. Except for descriptive statistics, those with missing data were not included in the analysis. Finally, data from 627 participants were analyzed (valid response rate = 6.9%). Participants’ demographic characteristics are presented in Table 1.

**Table 1:** Participants’ sociodemographic characteristics

Variables	n/(Mean)	%/[SD]
Gender		
Women	598	95.4
Men	29	4.6
Age (Years)	(41.4)	[9.5]
Period of working as a clinical nurse (Years)	(16.7)	[9.3]
Period of working as a home healthcare nurse (Years)		
<1	141	22.5
<2	245	39.1
<3	234	37.3
Missing	7	1.1
Work status		
Full-time	491	78.3
Part-time	135	21.5
Missing	1	0.2
Family constitution		
Alone	84	13.4
Living with families requiring caregiving or having underage children	342	54.5
Living separately from families requiring caregiving or having underage children	198	31.6
Missing	3	0.5

### Item analysis

Six initial items with ceiling effects were removed. Through the confirmation of the floor effect, item-total correlation coefficients (r < 0.3), and the G-P analysis, no item was deleted.

### Reliability analysis

Cronbach’s alphas ranged 0.897 to 0.941 for the four subscales and Cronbach’s alpha was 0.961 for the overall scale (Table 2). The ICC values in the test-retest survey completed by 32 nurses (response rate = 3.5%) ranged 0.703 to 0.905 for the four subscales and was 0.836 for the overall scale.

**Table 2:** Item analysis and exploratory factor analysis of the ESA-NHHN (n=627)

Items of the ESA-NHHN(α=0.96)and subscales with Cronbach’s α coefficient		mean	SD	ceiling effect	floor effect	Factor loading^a^			
Item						1	2	3	4
**Subscale 1: Acquire new knowledge(α=0.94)**									
1	On entering employment, there is the opportunity to learn about the system, including public healthcare insurance and government-based long-term care insurance.	3.23	1.17	4.40	2.06	**0.880**	-0.032	-0.103	-0.013
2	On entering employment, there is the opportunity to undergo training in the compensation structure for home healthcare nursing and users’ responsibilities for fees.	3.01	1.20	4.21	1.81	**0.946**	-0.066	-0.121	-0.049
3	On entering employment, there is the opportunity to receive instruction on the manners and conduct appropriate for home healthcare nurses.	3.38	1.12	4.50	2.27	**0.766**	0.015	-0.090	0.049
4	On entering employment, there is the opportunity to undergo training about emergency responses and contact methods at patients’ homes.	3.15	1.12	4.27	2.03	**0.834**	0.013	-0.046	-0.001
5	On entering employment, there is the opportunity to learn about an overview of the workplace, appropriate policies, safety-related precautions, and confirmation of the work schedule.	3.46	1.04	4.50	2.42	**0.675**	0.153	-0.063	0.073
6	On entering employment, there is the opportunity to undergo training about appropriate actions in the event of disasters.	2.81	1.17	3.98	1.65	**0.799**	-0.102	0.058	0.023
7	On entering employment, there is the opportunity to receive information about the location of the workplace, and local health, medical and welfare resources.	2.77	1.12	3.89	1.65	**0.780**	-0.043	0.052	0.049
8	On entering employment, there is the opportunity to receive information about the roles of other institutions and members of numerous professions.	3.15	1.08	4.23	2.07	**0.695**	0.097	0.001	0.028
9	Within the workplace, there are regular opportunities for training and participation in study groups.	3.74	1.04	4.78	2.70	**0.401**	0.090	0.311	-0.139
10	There are opportunities to undergo training for responding to users’ and family members’ dissatisfaction and complaints.	2.86	1.08	3.94	1.78	**0.672**	-0.033	0.124	0.029
11	There are opportunities for training in taking the protection of users’ and family members’ privacy into consideration when handling information and records.	3.18	1.08	4.26	2.10	**0.717**	0.019	0.103	-0.057
12	Before being responsible for terminal-stage users, there is the opportunity to undergo training about dealing with deaths at their homes.	2.83	1.11	3.94	1.72	**0.580**	0.054	0.247	-0.131
**Subscale 2: Concrete experience (α=0.90)**									
13	Experience can be gained in understanding the provision of care as a team, together with colleagues and managers.	3.97	0.86	4.83	3.11	0.148	**0.468**	0.068	0.130
14	The 24-hour response system includes a mechanism for obtaining support from colleagues promptly when needed.	3.94	1.02	4.96	2.92	0.020	**0.526**	-0.035	0.160
15	There are opportunities to understand that the consideration and manners needed when visiting the homes of home-care patients are different from those of a hospital nurse.	4.01	0.89	4.90	3.12	0.246	**0.595**	-0.206	0.138
16	One can repeatedly accompany colleagues on visits until one has gained sufficient confidence.	3.70	1.12	4.82	2.58	0.138	**0.417**	0.042	0.174
17	There are opportunities to learn in practice how the status of the use of sanitary materials differs between hospital and home healthcare nurses.	4.10	0.84	4.94	3.26	-0.049	**0.902**	-0.078	-0.121
18	Newly appointed home healthcare nurses have opportunities to talk about their own thoughts, feelings, and experiences and speak at meetings for responsible personnel and so on, attended by members of multiple professions.	3.47	1.06	4.53	2.41	0.111	**0.539**	0.274	-0.174
19	There are opportunities to appreciate the importance of information exchange with members of other professions.	4.16	0.77	4.93	3.39	-0.062	**0.778**	-0.032	-0.052
20	There are opportunities to share one’s own experience of solo visits with colleagues and others.	4.12	0.81	4.93	3.31	-0.123	**0.590**	0.248	0.002
21	There are places and environments that enable the provision of nursing care that prioritizes the values and perspectives about the life and death of home-care patients and their family members.	3.74	0.88	4.62	2.86	0.007	**0.632**	0.101	0.073
22	There are opportunities to learn about the importance of verbally expressing appreciation to family members who support home-care patients.	4.04	0.81	4.85	3.23	-0.022	**0.732**	-0.065	0.092
**Subscale 3: Reflective observation of own experience (α=0.89)**									
23	At case-study meetings, there are opportunities to reflect on what one has learned oneself and to integrate this with what other people have learned.	3.38	1.07	4.45	2.31	0.169	0.144	**0.586**	-0.128
24	There are opportunities to reflect on daily care, using checklists, and others, together with one’s colleagues.	3.16	1.13	4.29	2.03	0.185	-0.117	**0.587**	0.126
25	There are regular opportunities for face-to-face meetings with colleagues.	3.34	1.15	4.49	2.19	-0.004	-0.093	**0.799**	0.083
26	There are opportunities to reflect on one’s own nursing home visits by means of discussion with colleagues.	3.76	0.94	4.70	2.82	-0.203	0.234	**0.666**	0.042
27	There are opportunities to reflect on one’s own experience at conferences and thus to fully appreciate the significance of that experience.	3.51	1.05	4.56	2.46	-0.024	0.049	**0.860**	-0.060
28	There is support for setting targets for specific and realistic practice by newly appointed home healthcare nurses.	3.23	1.10	4.33	2.13	0.237	-0.084	**0.580**	0.158
**Subscale 4: Support from home healthcare agency managers (α=0.92)**									
29	There is the potential to discuss with colleagues and/or managers the stress felt in connection with home healthcare nursing activities.	3.85	0.95	4.80	2.90	-0.123	0.264	0.176	**0.419**
30	Colleagues and/or managers form connections with newly appointed home healthcare nurses to enable the nurses to develop a strong ability to look carefully at and think about their daily experiences.	3.59	0.97	4.56	2.62	0.048	0.185	0.215	**0.489**
31	There are opportunities for regular face-to-face consultations with managers.	3.64	1.13	4.77	2.51	0.035	-0.162	0.465	**0.450**
32	Managers provide newly appointed home healthcare nurses feedback about their work as a whole, as appropriate to the circumstances.	3.85	0.99	4.84	2.86	-0.080	0.101	0.070	**0.808**
33	When newly appointed home healthcare nurses enter employment, managers give them advice about ways to respond to problems with which they have considerable experience.	3.76	1.05	4.81	2.71	0.043	0.032	-0.006	**0.853**
34	Managers respond on an individual level to newly appointed home healthcare nurses, taking each nurse’s background into consideration.	3.82	1.03	4.85	2.80	-0.009	0.081	-0.056	**0.886**

Bold figures indicate factor loading >0.4

a:Factor loadings were based on exploratory factor analysis with maximum likelihood method and Promax rotation

The ESA-NHHN:The Educational Support Assessment Scale for Novice Home Healthcare Nurses

### Validity analysis

**Fig. 2:**
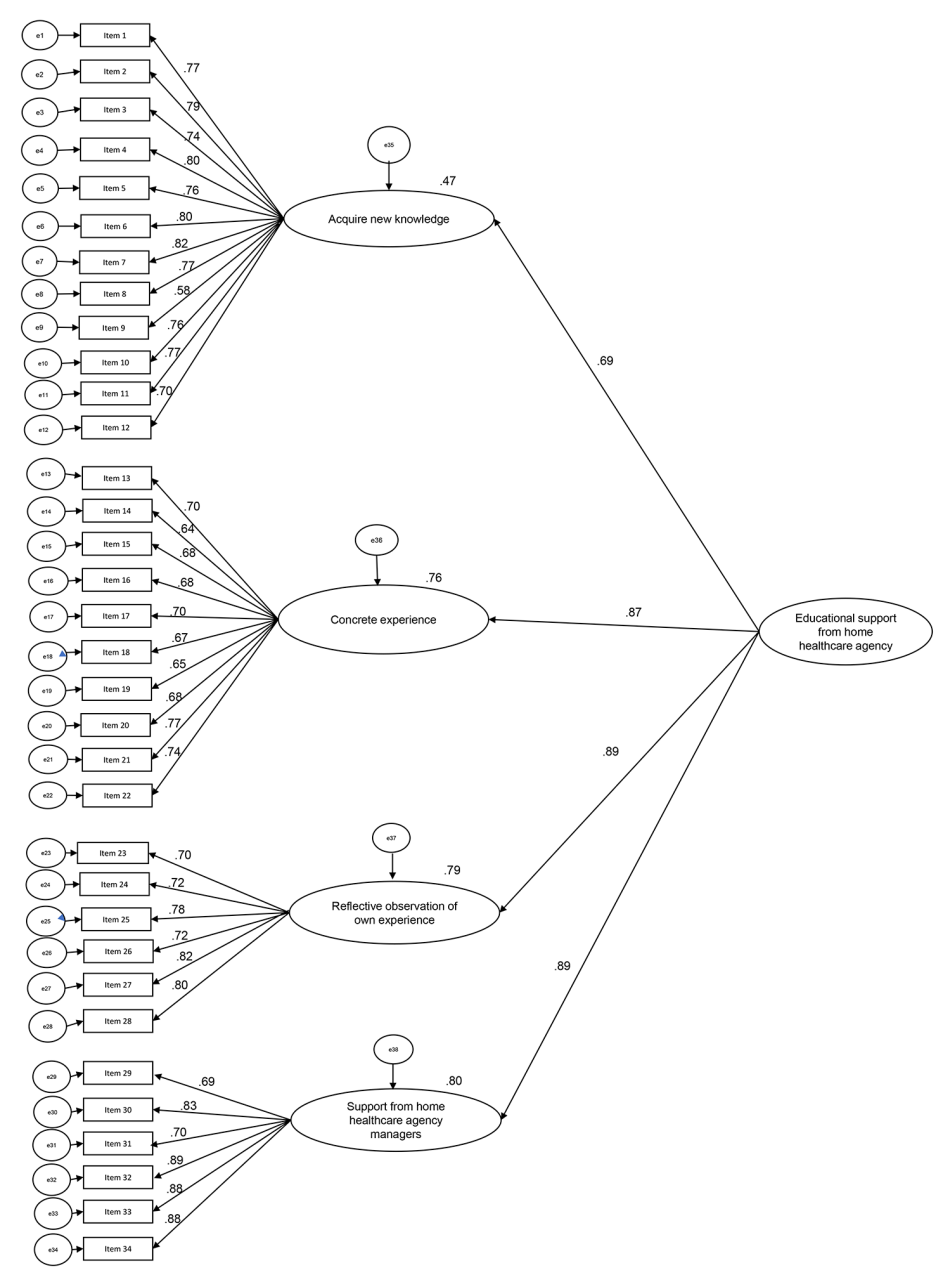
Results of the confirmatory factor analysis of the ESA-NHHN

The KMO score was 0.964, and Bartlett’s chi-square test of sphericity was significant (χ^2^ = 14 155.3, df = 528, p < 0.001). Thus, the EFA was appropriate. The EFA generated a four-subscale structure consisting of 34 items, which supported the hypothesized components we assumed (Table 2). Model fit indices of the CFAs were as follows: CFI = 0.876, GFI = 0.810, TLI = 0.867, and RMSEA = 0.076 [90% CI: 0.072–0.079] (Fig. 2).

The scores of the ESA-NHHN were significantly positively correlated with psychological variables such as affective commitment (Table 3). Concurrent validity was confirmed.

**Table 3:** Partial correlation coefficients between the total and each ESA-NHHN subscale with related scales (n=627)

Control variable	Scales	Affective commitment	Work engagement	HHNJS-J	Satisfaction with their agencies-1	Satisfaction with their agencies-2
Age	The ESA-NHHN total	0.540**	0.375**	0.595**	0.742**	0.608**
	Subscale 1: Acquire new knowledge	0.412**	0.274**	0.421**	0.629**	0.396**
	Subscale 2: Concrete experience	0.498**	0.354**	0.595**	0.608**	0.561**
	Subscale 3: Reflective observation of one’s own experience	0.457**	0.326**	0.517**	0.642**	0.505**
	Subscale 4: Support from home healthcare agency managers	0.517**	0.366**	0.567**	0.668**	0.728**

The ESA-NHHN:The Educational Support Assessment Scale for Novice Home Healthcare Nurses

HHNJS-J:Japanese version of the Home Healthcare Nurses’ Job Satisfaction Scale

Satisfaction with their agencies-1: “I feel I received educational support in adjusting to home healthcare at this home healthcare agency.”

Satisfaction with their agencies-2: “I feel that the manager of this healthcare agency is a good manager and leader.”

**p<0.01

## Discussion

This study developed and verified the reliability and validity of the ESA-NHHN, which measures educational support for novice home healthcare nurses. The results indicated adequate internal consistency, test-retest reliability, structural validity, and concurrent validity.

The internal consistency was acceptable. Cronbach’s alpha of the overall ESA-NHHN was 0.961, and Cronbach’s alphas for the four subscales ranged 0.889 to 0.941, indicating good to excellent reliability for the newly developed scale. Regarding test-retest reliability, the ICC values were ≥ 0.703, confirming high stability.

Regarding structural validity, a four-subscale structure was extracted using the EFA. The final scale comprises 34 items distributed into four subscales: “acquire new knowledge,” “concrete experience,” “reflective observation of own experience,” and “support from home healthcare agency managers.” The relevance of these four subscales was compatible with hypothetical components. CFA, to test the fitness of the data to the subscale structure, revealed a CFI value of 0.876 and a GFI value of 0.810, which was slightly below the recommended value of 0.90; however, the RMSEA value was 0.076, which was a reasonable fit. Overall, the structural validity of the four-subscale structure of the ESA-NHHN was moderately acceptable.

Regarding concurrent validity, partial correlation coefficients showed a significant positive relationship between the total score and the four subscales of the ESA-NHHN and affective commitment, work engagement, job satisfaction of home healthcare nurses, and satisfaction with their home healthcare agencies (Table 3). The significant positive correlations found between these concepts and the ESA-NHHN indicate that home healthcare agencies where educational support is provided (as confirmed by the ESA-NHHN items) are more likely to foster a higher sense of belonging and job satisfaction among home healthcare nurses. Therefore, concurrent validity indicated acceptability.

### Limitations

This study had several limitations. First, selection bias is possible, with a response rate of 8.1%, which is considerably low. There could be two reasons for this. First, because the number of applicable respondents in each agency could have been small owing to the many criteria as novice home healthcare nurses, the response rate could have been estimated as smaller than the actual percentage. Nurses’ years of clinical experience at each home healthcare agency was not made public. Therefore, we could not confirm in advance whether there were applicable respondents at each agency. As surveys were mailed randomly, it is possible that the survey was sent to agencies without applicable respondents. Second, the survey could have been conducted when the settings were overwhelmed because of the spread of coronavirus disease 2019. Further, the ESA-NHHN was developed in Japan, and we cannot easily compare the current results with those found in other countries. In the future, the English version of the ESA-NHHN should be evaluated in different settings and among participants from various countries to confirm the generalizability of the scale.

### Implications for nursing practice

Implementing the ESA-NHHN could help novice home healthcare nurses settle into the field and prevent turnover. The ESA-NHHN enables managers of home healthcare agencies to evaluate and visualize the educational support provided by novice home healthcare nurses and could be a clue to improving the support provided by their agencies.

## Conclusions

The ESA-NHHN was developed based on a comprehensive literature review, including a reference to Kolb’s experimental learning theory. Experimental learning theory emphasizes that the learning process occurs as a result of the integration of concrete emotional experiences with cognitive processes [37]. This study developed a reliable and valid scale—the ESA-NHHN—to assess the educational support for novice home healthcare nurses. Managers in home healthcare agencies should apply the results of assessments using the ESA-NHHN to improve their human resource development.

## Electronic supplementary material

Below is the link to the electronic supplementary material.

Supplementary Material 1

## Data Availability

The datasets generated and analyzed during this study are available from the corresponding author on reasonable request.

## Abbreviations

DREEM: Dundee Ready Education Environment Measure
ESA-NHHN: Educational Support Assessment Scale for Novice Home Healthcare Nurses
CVI: content validity index
EFA: exploratory factor analysis
HHNJS-J: Japanese version of the Home Healthcare Nurses’ Job Satisfaction Scale
G-P analysis: good-poor analysis
ICC: intraclass coefficient
KMO: Kaiser-Meyer-Olkin index
CFA: confirmatory factor analysis
GFI: goodness-of-fit index
CFI: comparative fit index
TLI: Tucker-Lewis index
RMSEA: root mean square error of approximation
90% CI: 90% confidence interval
