# Supplementary material for: Development and psychometric validation of an educational support assessment scale for novice home healthcare nurses
Source: BMC Med Educ. 2023 May 10;23:326. doi: 10.1186/s12909-023-04313-1 (PMC10171149; doi:10.1186/s12909-023-04313-1)
Supplement: Supplementary file 1 — Supplementary Material 1 [file 12909_2023_4313_MOESM1_ESM.docx]

Additional file 1: Search Formulas used in the literature review

Data Bases: MEDLINE (Ovid), CINAHL, ICHUSHI (in Japanese)

Search Formulas:

“home health nursing”

“home health nursing” AND “novice nurs*”

“home healthcare” AND education AND training

“home healthcare” AND reflection

“home healthcare” AND feedback

“home healthcare” AND “training of novice nurs*”

“home healthcare” AND re-learning

“home healthcare” AND “human resource development”
